# Electronic metal-support interactions in vacuum vs. electrolyte

**DOI:** 10.1038/s41467-020-15306-9

**Published:** 2020-03-19

**Authors:** Tobias Binninger

**Affiliations:** IBM Research – Zurich, Säumerstrasse 4, CH-8803 Rüschlikon, Switzerland

**Keywords:** Catalysis, Electrocatalysis, Surface chemistry, Electrochemistry

**Arising from** C. Jackson et al. *Nature Communications* 10.1038/ncomms15802 (2017).

Electronic metal-support interactions (EMSI)^1–5^ describe the electron transfer between metal catalyst nanoparticles and their supporting material due to electronic equilibration. Recently, Kramer and co-workers^6^ reported the experimental observation of an inversion of the relative sign of EMSI-related charge transfer in an electrochemical environment compared to vacuum, which they explained with a well-known relation between work function (The work function of a material surface is the minimum energy required to extract an electron therefrom.) and potential of zero charge (PZC) (The PZC is the potential of a metal electrode where it carries zero surface charge—for greater potentials, the metal surface carries a positive charge, and vice versa.) of metal surfaces^7^. From my perspective, this explanation has to be taken with care, because this work function–PZC relation considers metal surfaces that are uncharged in vacuum, and it therefore does not directly apply to EMSI. A careful analysis both of the electrostatic boundary conditions and of the spatial distribution of the transferred electronic charge on the catalyst nanoparticle reveals that electrochemical EMSI inversion is expected to be observable only under special circumstances.

Kramer and co-workers^6^ experimentally observed higher intrinsic oxygen reduction reaction activity of Pt nanoparticles supported on graphite-rich boron carbide (Pt/BC) compared to Pt supported on carbon black (Pt/C), which they attributed to different EMSI: A stronger transfer of electrons from the support to the Pt nanoparticles was postulated for Pt/BC compared to Pt/C based on an interpretation of X-ray photoelectron spectroscopy (XPS) data obtained in ultrahigh vacuum (UHV). The authors explained that a smaller BC support work function (compared to C) would result in stronger electron transfer to Pt and, thus, more negatively charged Pt nanoparticles. In contrast, in situ X-ray absorption spectroscopy (XAS) performed in acidic electrolyte under potential control revealed more positively charged Pt nanoparticles on the BC support compared to Pt/C. This apparent contradiction between the relative sign of the Pt charge observed in XPS and XAS experiments was tentatively ascribed to the different environments: XPS in UHV vs. in situ XAS in electrolyte under potential control. The inverted relative sign of Pt charging in the latter environment was explained with the proportionality between Pt work function and its PZC with reference to ref. ^7^: Kramer et al. argued that Pt on BC support would have a smaller work function which would negatively shift its PZC and, thus, cause a more positive charging of the Pt nanoparticle compared to Pt on C support in an electrolyte at the same electrode potential.

In this correspondence, I would like to show that the support-induced shift of the Pt nanoparticle work function in vacuum cannot be used to infer a corresponding shift of the Pt PZC in an electrochemical environment. Indeed, the PZC of a metal electrode is approximately linearly dependent on the metal work function as expressed in Relation (8) of ref. ^7^ or (9) of ref. ^8^. But here, the work function of an uncharged homogeneous metal surface in vacuum is meant. Charge redistribution between support material and Pt nanoparticle on a heterogeneous surface is not taken into account. Instead, it is demonstrated in ref. ^5^ that the EMSI-related Pt nanoparticle charging produces additional electrostatic fields that extend away from the surface. These fields result in an additional electrostatic potential contribution that shifts the work function of the supported Pt nanoparticle. Thus, the support-induced Pt nanoparticle charging observed in UHV is the cause and the Pt work function shift is the effect. This Pt work function shift cannot be inserted into Relation (8) of ref. ^7^. Doing so, the Pt work function shift would translate into a shift of the Pt PZC, which, in turn, would cause an inversion of the Pt nanoparticle charging: The effect would invert its own cause.

This contradiction can be resolved by adapting the derivation in ref. ^7^ to the present situation of a supported Pt nanoparticle. For clarity of presentation, we assume the same work function for all facets of the Pt nanoparticle (Strictly speaking, the work function differs between different facets of the nanoparticle, which results in an additional charge redistribution across the Pt nanoparticle surface^9^ in addition to EMSI-induced charge transfer from the support. These facet effects are closely related to the EMSI effects under consideration in the present correspondence, and a similar analysis holds for the influence of the environment on such facet charge redistribution.). We first consider the case of a small width *w*_dl_ of the electrochemical double layer compared to the size *d*_Pt_ of the Pt nanoparticle, *w*_dl_ ≪ *d*_Pt_. This condition is fulfilled in relevant electrocatalysis settings, as encountered in the study of Kramer et al.: For aqueous electrolyte concentrations ≥0.1 M, the dominant part of the electrochemical double layer lies in the Stern layer^10–12^ within the outer Helmholtz plane distance *d*_OHP_ from the electrode surface (*d*_OHP_ is the distance of closest approach of hydrated ions to the electrode surface, and it is approximately equal to the radius of hydrated ions plus one water layer^12^.), and *d*_OHP_ ≈ 0.6 nm for anions such as ClO${}_{4}^{-}$ ref. ^13^, thus fulfilling *w*_dl_ ≪ *d*_Pt_ for *d*_Pt_ > 2 nm. In such case, both the electrolyte and the bulk metal of the Pt nanoparticle strongly screen the external Pt nanoparticle surface from the EMSI-induced charge transfer, and the latter is largely confined to a narrow double layer at the direct support–Pt interface, as schematically shown in Fig. 1b. Therefore, at the PZC ${E}_{{q}_{{\rm{Pt}}} = 0}^{{\rm{Pt}}/{\rm{Sup}}}$, i.e. for zero charge on the *external* Pt nanoparticle surface in contact with the electrolyte, the potential differences add up along the path indicated in Fig. 1a in the same way as in ref. ^7^,

1 $${E}_{{q}_{{\rm{Pt}}} = 0}^{{{\rm{Pt}}} {/} {{\rm{Sup}}}} =	 \,{\it{\Phi}}_{{\rm{Sup}}}-{\it{\Phi}}_{{\rm{Sup}},{\rm{Ref}}}\\ =	 \,{}^{{\rm{Sup}}}{\Delta }^{{\rm{Pt}}}{\it{\Phi}} {\,\,}{+}{\,\,}^{{\rm{Pt}}}{\Delta }^{{\rm{sol}}}{\it{\Phi}}_{{q}_{{\rm{Pt}}} = 0}{\,\,}{+}{\,\,}^{{\rm{sol}}}{\Delta }^{{\rm{M}},{\rm{Ref}}}{\it{\Phi}} {\,\,}{+}{\,\,}^{{\rm{M}},{\rm{Ref}}}{\Delta }^{{\rm{Sup}},{\rm{Ref}}}{\it{\Phi}} \\ =	\, {}^{{\rm{Pt}}}{\Delta }^{{\rm{sol}}}{\it{\Phi}}_{{q}_{{\rm{Pt}}} = 0}{\,\,}{-}{\,\,}^{{\rm{M}},{\rm{Ref}}}{\Delta }^{{\rm{sol}}}{\it{\Phi}} {\,} + {\,} \frac{1}{F}\ ({\mu }_{{\rm{e}}}^{{\rm{M}}}-{\mu }_{{\rm{e}}}^{{\rm{Pt}}}).$$

Here, *Φ*_i_ is the inner electrostatic potential of phase i and the notation from ref. ^7^ was used, i.e. ^A^Δ^B^*Φ* = *Φ*_A_− *Φ*_B_, and ${\mu }_{{\rm{e}}}^{i}$ denotes the chemical potential of electrons in phase i. Furthermore, electronic equilibration across the two metal-support interfaces was assumed, i.e. ${}^{{\rm{Sup}}}{\Delta }^{{\rm{Pt}}}{\it{\Phi}} =\frac{1}{F}\ ({\mu }_{{\rm{e}}}^{{\rm{Sup}}}-{\mu }_{{\rm{e}}}^{{\rm{Pt}}})$ and ${}^{{\rm{M}},{\rm{Ref}}}{\Delta }^{{\rm{Sup}},{\rm{Ref}}}{\it{\Phi}} =\frac{1}{F}\ ({\mu }_{{\rm{e}}}^{{\rm{M}}}-{\mu }_{{\rm{e}}}^{{\rm{Sup}}})$. Expression (1) is identical to the corresponding Equation (2) of ref. ^7^. Because the latter was derived for an extended, unsupported Pt metal electrode, it follows that for *w*_dl_ ≪ *d*_Pt_, the PZC at most of the external surface of Pt nanoparticles remains unaffected by the support. Therefore, in electrolytes with a short screening length, EMSI charge transfer is largely confined to the direct support–Pt interface, which is inaccessible for the electrolyte. In particular, no inversion of the relative charge transfer is deduced.

**Fig. 1:**
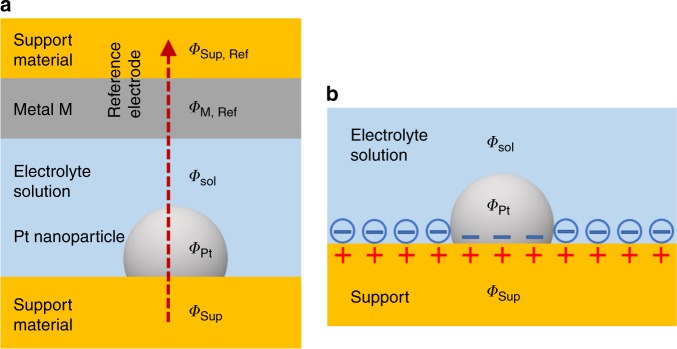
Electrostatic conditions around a supported Pt nanoparticle in electrochemical environment. **a** Schematic cell used to derive Eq. (1) with Pt/Support electrode, electrolyte, and reference electrode (Ref). For ease of derivation and without loss of generality, the reference electrode metal M is considered to form a dense layer on a substrate of the same support material. The inner electrostatic potentials of different phases i are denoted by *Φ*_i_. **b** Interface charging at the Pt/Support electrode in a strongly screening electrolyte, i.e. for *w*_dl_ ≪ *d*_Pt_, at the PZC ${E}_{{q}_{{\rm{Pt}}} = 0}^{{\rm{Pt}}/{\rm{Sup}}}$, i.e. for zero charge on the *external* Pt nanoparticle surface in contact with the electrolyte. Negative ions of the electrolyte are depicted by circles with negative signs inside. EMSI charge transfer is confined to the direct support–Pt interface, and it closes the “gap” in the electrochemical double layer of the support at the place of the Pt nanoparticle. The external Pt nanoparticle surface is screened by the strong electrolyte from the support interface, and thus its PZC is the same as for an unsupported Pt electrode.

In the opposite case of a weakly screening electrolyte, i.e. *w*_dl_ ≫ *d*_Pt_, the plateau value of *Φ*_sol_ in the electrolyte is reached at a distance much greater than the size of the Pt nanoparticle. The charge and electrostatic field of the electrochemical double layer become small around the Pt nanoparticle in comparison to the charge and fields originating from electronic equilibration between Pt and support. Consequently, the latter are mainly affected by the dielectric properties of the solvent surrounding the Pt nanoparticle as explained in ref. ^5^, which can enhance EMSI-related charge transfer to the external Pt nanoparticle surface, but which cannot invert its direction.

So far, the support work function was considered to be constant. However, electrochemical processes at the support surface can change the support work function in an electrochemical environment^14,15^. Such changes do not affect the boundary conditions for the electrostatic potential distribution: Firstly, the Galvani potential step ${}^{{\rm{Sup}}}{\Delta }^{{\rm{sol}}}{\it{\Phi}}$ between support and electrolyte remains fixed by the external potential control. Therefore, any change of the support work function due to electrochemical processes must be compensated by an opposite change in the electrochemical double layer at the support surface. Secondly, the potential step at the direct support–Pt interface remains fixed by the requirement of electronic equilibration, i.e. ${}^{{\rm{Sup}}}{\Delta }^{{\rm{Pt}}}{\it{\Phi}} =\frac{1}{F}\ ({\mu }_{{\rm{e}}}^{{\rm{Sup}}}-{\mu }_{{\rm{e}}}^{{\rm{Pt}}})$. Therefore, the above analysis for the case *w*_dl_ ≪  *d*_Pt_ still holds: The external Pt nanoparticle surface is screened from electrochemical processes at the support surface and the EMSI charge transfer remains fixed at the direct support–Pt interface. Conversely, for *w*_dl_ ≥ *d*_Pt_, an electrochemical change of the support work function can affect the charging of the external Pt nanoparticle surface and potentially also its sign. Still, the EMSI charge transfer at the direct support–Pt interface remains fixed.

Finally, it is important to note that both XPS and XAS reveal an average charge state of the Pt nanoparticle as a whole. Therefore, for an electrochemical inversion of relative EMSI charge transfer to be detectable by in situ XAS, (1) the relative support workfunction must be inverted due to electrochemical processes, (2) the electrolyte must be dilute, i.e. weakly screening, and (3) the resulting EMSI effect at the external Pt nanoparticle surface must dominate over the unaffected EMSI charging at the direct support–Pt interface.

## Data Availability

No datasets were generated or analysed during the current study. Any further information related to this work is available from the authors upon reasonable request.
